# The pre-ablation triglyceride-glucose index predicts late recurrence of atrial fibrillation after radiofrequency ablation in non-diabetic adults

**DOI:** 10.1186/s12872-022-02657-y

**Published:** 2022-05-14

**Authors:** Qinghui Tang, Xiao-Gang Guo, Qi Sun, Jian Ma

**Affiliations:** Arrhythmia Center, State Key Laboratory of Cardiovascular Disease, Fuwai Hospital, National Center for Cardiovascular Diseases, Chinese Academy of Medical Sciences and Peking Union Medical College, 167 Bei Li Shi Road, Xicheng District, Beijing, 100037 China

**Keywords:** Atrial fibrillation, Triglyceride-glucose index, Biomarkers, Predictors of rhythm outcome, Catheter ablation

## Abstract

**Background:**

Current prognostic risk scoring systems and biomarkers are routinely used as non-invasive methods for assessing late recurrence of atrial fibrillation (AF) in patients who have undergone radiofrequency catheter ablation (RFCA). This study aimed to investigate the predictive value of the triglyceride-glucose (TyG) index for late AF recurrence after RFCA in non-diabetic patients.

**Methods:**

In total, 275 patients with AF who underwent RFCA at the Fuwai hospital (Beijing, China) between January 2016 and December 2018 were enrolled in this study. During follow up, patients were divided into late and non-late AF recurrence groups, based on whether they had experienced late AF recurrence determined by electrocardiography (ECG) examine or 48 h Holter monitoring. The TyG index was calculated using the following equation: ln [fasting triglycerides [mg/dL]** × **fasting glucose [mg/dL]/2].

**Results:**

During a median follow-up of 26.1 months, late AF recurrence event rates significantly increased in the highest TyG index tertile group (tertile 3) compared to the lowest group (tertile 1) (54% versus 12%, respectively; *p* < 0.001). The mean TyG index was higher in the late AF recurrence group compared to the non- late AF recurrence group (9.42 ± 0.6 versus 8.68 ± 0.70, respectively; *p* < 0.001). On multivariate Cox regression analysis, the pre-ablation TyG index was an independent risk factor for late recurrence of AF after RFCA (hazard ratio [HR] 2.015 [95% confidence interval (CI): 1.408–4.117]; *p* = 0.009). Receiver operating characteristic (ROC) curve analysis revealed that TyG index was a significant predictor of late AF recurrence after RFCA, with an area under the ROC curve (AUC) of 0.737 (95% CI: 0.657–0.816; *p* < 0.001). In addition, the AUC of left atrial diameter (LAD) was 0.780 (95%CI: 0.703–0.857, *p* < 0.001). Finally, the TyG index positively correlated with LAD (r = 0.133, *p* = 0.027), high sensitivity C-reactive protein (r = 0.132, *p* = 0.028) and N-terminal pro B-type natriuretic peptide (r = 0.291, *p* < 0.001) levels.

**Conclusions:**

An elevated pre-ablation TyG index was associated with an increased risk of late AF recurrence after RFCA in non-diabetic patients. The TyG index may be potentially useful as a novel biomarker for the risk stratification of late AF recurrence in non-diabetic patients.

## Background

Atrial fibrillation (AF) is an extremely costly public health issue [1]. Worldwide, radiofrequency catheter ablation (RFCA) is the gold standard therapy for drug-refractory AF [2, 3]. Nevertheless, the long-term AF-free survival period without atrial arrhythmia recurrence remains unsatisfactory, with a reported late AF recurrence rate of up to 30–50% after first pulmonary vein (PV) isolation [4, 5]. Because of the high recurrence rates after RFCA, it is crucial to investigate the clinical risk variables that influence the successful maintenance of sinus rhythm in patients with AF after RFCA [6]. The treatment of patients with recurrent AF after RFCA is challenging [4, 7, 8]. Moreover, among patients with a high risk of post-ablation AF recurrence or with multiple atrial structural remodeling, the cost-effectiveness of RFCA is low [7, 8]. Although several scoring systems, such as the APPLE [Age > 65 years, Persistent AF, impaired eGFR [< 60 ml/min/1.73 m ^2^], Left atrial diameter ≥ 43 mm, Left ventricular ejection fraction < 50%] [9], CHA_2_DS_2_-VASc[congestive heart failure, hypertension, age ≥ 75, diabetes, stroke, vascular disease, age 65 to 74 and sex category] [10], and DR-FLASH score [based on Diabetes mellitus, Renal dysfunction, Persistent AF type, LA diameter > 45 mm, Age > 65 years, Female Sex, and Hypertension])[11], and several biomarkers have recently been proposed to predict late AF recurrence after RFCA, there is no clear consensus regarding a risk scoring system or biomarkers predicting rhythm outcome(s) after RFCA [9–11].

Experimental and clinical findings have revealed that abnormal glucose metabolism and insulin resistance (IR) are involved in atrial electrical and structural remodeling processes and AF development [12, 13]. The triglyceride glucose index (TyG index), calculated using fasting blood glucose and triglyceride (TG) levels, is considered to be a reliable index of IR in clinical practice [13, 14]. IR is a pathological state in which cells or tissues cannot respond normally to insulin, and is characterized by increased body weight, hyperglycemia, dyslipidemia, and elevated blood pressure [15, 16]. Cardiometabolic syndrome and IR are also common among non-diabetic patients and are associated with epicardial fat deposition and vulnerability to AF [12, 17, 18]. IR can induce AF by increasing left atrial (LA) volume or impairing left ventricular diastolic function [18]. However, the prognostic value of the TyG index in patients with late AF recurrence after RFCA remains controversial. This study aimed to validate the TyG index for predicting the rhythm outcome of non-diabetic AF patients who underwent RFCA.

## Methods

### Study population

This retrospective study enrolled 380 consecutive non-diabetic patients who underwent first-time radiofrequency catheter ablation for AF between January 2016 and December 2018 at Fuwai Hospital (Beijing, China). Patients with AF episodes lasting > 7 days were defined as having persistent AF, and those whose episodes terminated spontaneously within 7 days were defined as having paroxysmal AF. Long-standing persistent AF was defined as persistent AF episodes lasting > 12 months [1]. Obesity was defined as a body mass index (BMI) > 28 kg/m^2^ according to the criteria recommended by the National Health Commission in China [19]. Patients with secondary AF (e.g., related to infection, heart surgery, or hyperthyroidism); acute coronary syndrome requiring interventional management; ischemic cardiomyopathy; contraindications to anticoagulation; decompensated heart failure (baseline NYHA classification of class III-IV); left atrial thrombosis, moderate to severe valvular diseases, any taking antidiabetic or antihyperlipidemic agents at baseline and pregnancy, were excluded. The study was approved by the Ethics Committee of Fuwai Hospital, National Center for Cardiovascular Diseases, Chinese Academy of Medical Sciences, and Peking Union Medical College, and was performed in accordance with the Helsinki Declaration. Written informed consent was obtained from all patients.

### Risk scoring system

As described above, the APPLE score was evaluated based on 1 point for age > 65 years, persistent AF type, impaired estimated glomerular filtration rate (< 60 ml/min/1.73m^2^), LA diameter > 43 mm, left ventricular ejection fraction < 50%; it ranged from 0 to 5 points, and was assessed before the procedure [9]. The DR-FLASH score was assessed based on 1 point for each of the following parameters: diabetes mellitus, renal dysfunction, LA diameter > 45 mm, persistent AF type, age > 65 years, female sex, and hypertension, and ranged from 0 to 7 points [11].The CHA_2_DS_2_-VASc, APPLE, and DR-FLASH scores were calculated for each patient before AF ablation.

### Blood sample collection and processing

Before ablation, fasting blood samples were collected from all patients within 24 h of admission. Serum blood biomarkers (fasting plasma glucose and lipid parameters, including total cholesterol, TG, low density lipoprotein cholesterol (LDL-C) and high density lipoprotein cholesterol (HDL-C)) were assessed using standard laboratory techniques at Fuwai Hospital. The TyG index was calculated using the following equation: ln [fasting TG [mg/dL] × fasting glucose [mg/dL]/2].

### Electrophysiological study and catheter ablation

Radiofrequency catheter ablation was performed under deep sedation; electrocardiography and oxygen saturation were monitored continuously during the procedure. LA access was established under X-ray fluoroscopic guidance using double trans-septal puncture. After assessment of the left atrium, a Lasso catheter (Biosense Webster, Diamond Bar, CA, USA) and a 3.5-mm-tip ablation catheter were advanced into left atrium using 8 Fr SL1 trans-septal sheaths (St. Jude Medical Inc., St. Paul, MN, USA). After trans-septal access, a 100 UL/kg bolus of heparin was administered intravenously to maintain an activated clotting time of 300–350 s during the ablation procedure. Three-dimensional electroanatomic mapping of the left atrium was performed using the CARTO 3 system (CARTO, Biosense Webster Inc., Diamond Bar, CA, USA) and the point-by-point method. The PV ostium was identified by selective venography and tagged on the CARTO 3 electroanatomic map. A Lasso catheter was placed approximately 0.5 cm within the PV ostium for real-time monitoring of PV isolation. A contact force of 10–20 g was applied to each ablation point. RFCA energy was delivered at a power setting of 25–35 W with a maximal temperature of 43 °C for at least 30 s for each ablation point. During ablation, the infusion rate was 17–25 mL/min. The inter-lesion ablation point distance for PV isolation was mainly based on local electrogram mapping at the operator’s discretion. PV isolation was defined as PV entrance and exit block confirmed 30 min after the initial isolation with intravenous adenosine to examine acute PV reconnection. PV isolation was the only target and no additional ablation lines were performed. If non-PV focal triggers were documented, then they were ablated using an irrigated contact force RF catheter. If AF persisted after PV isolation, cardioversion was performed. If a common-type atrial flutter was documented, cavotricuspid isthmus linear ablation was performed to achieve bidirectional block.

### Post-procedural management

Transthoracic echocardiography was performed to exclude pericardial effusion after RFCA. Oral anticoagulants therapy was initiated the day after RFCA. Based on individual CHA_2_DS_2_-VASc scores, patients with AF continued to take oral anticoagulants for at least two months according to European Society of Cardiology Guidelines [1]. All patients with persistent AF continued to take amiodarone for three months after RFCA to maintain sinus rhythm and visited the doctors to monitor the side effects of the drug. For paroxysmal AF, any class I or III antiarrhythmic drug would be avoided if patients were free from AF recurrence during the three-month blanking period. In patients with AF recurrence, rate control with β-receptor blockers would first be considered, followed by propafenone as a second-line treatment, if β-receptor blockers did not alleviate the symptoms of discomfort. All class I or III antiarrhythmic drugs, if administered, were stopped at the end of the blank period.

### Follow-up

All patients were scheduled for routine outpatient follow up visits in our hospital at 1-, 3- and 12-months of the 1st year post-ablation, and every 12 months thereafter. Additionally, all patients were advised to receive 12-lead electrocardiography (ECG) or a 48-h Holter examination every month at their local hospital, and an additional 48-h Holter examination if prompted by recurrence of arrhythmia-related symptoms. Outpatient follow-up visits included history taking, physical examination, 12-lead ECG or 48 h-Holter monitoring, and echocardiography. Telephone interviews were conducted to screen for arrhythmia -related symptoms (chest discomfort, palpitations, fatigue, and dizziness). 12-lead ECG or 48-h Holter recording data were sent to the indicated electronic health record system. Early AF recurrence was defined as atrial arrhythmia recurrence within three-month blanking period. Late AF recurrence was defined as AF, atrial flutter, or atrial tachycardia lasting > 30 s recorded by any type of ECG or Holter monitoring after a three-month blanking period. The “AF-free” period was defined as no recurrence of atrial arrhythmia without antiarrhythmic drug use.

### Statistical analysis

Data distribution in this study was assessed using the Kolmogorov–Smirnov criterion. Continuous variables are expressed as mean ± standard deviation (SD) if normally distributed. Otherwise, they were expressed as medians (interquartile range). The study population was stratified into three tertiles according to the value of pre-ablation TyG index (tertile 1:6.80 < TyG index < 8.67; tertile 2:TyG index:8.68–9.37; and tertile 3:TyG index ≥ 9.38). Comparisons of two continuous variables with normal distribution in the study were performed using the independent samples Student’s t-test. Categorical variables are expressed as frequencies and percentages, and were compared using chi-squared test or Fisher’s exact test. Nonparametric variables were compared using the Wilcoxon signed-rank test. Analysis of ANOVA test was used to compare TyG index according to the APPLE, DR-FLASH and CHA_2_DS_2_-VASc scores. Univariate and multivariate regression analyses using the backward likelihood ratio method were used to determine the risk predictors of AF recurrence. The covariables in the multivariate Cox regression analysis were mainly selected for the following reasons: the variables were statistically significant in the univariate analysis, or the variables were known to be related to cardiovascular events and thus may serve as potential confounders. Correlation analyses were performed using Spearman’s rank test. Receiver operating characteristic (ROC) curve analysis was used to assess the predictive value of clinical factors for late AF recurrence and to identify the optimal cut-off value using Youden’s index. Kaplan–Meier survival curves were constructed to evaluate the incidence rate of late AF recurrence in each group, according to tertiles of TyG index. All data were analyzed using SPSS version 25.0 (IBM Corporation, Armonk, NY, USA) and GraphPad Prism version 5.0 (GraphPad Inc, San Diego, CA, USA). Differences were considered statistically significant at a two-tailed *P* < 0.05.

## Results

### Patients population

**Fig. 1:**
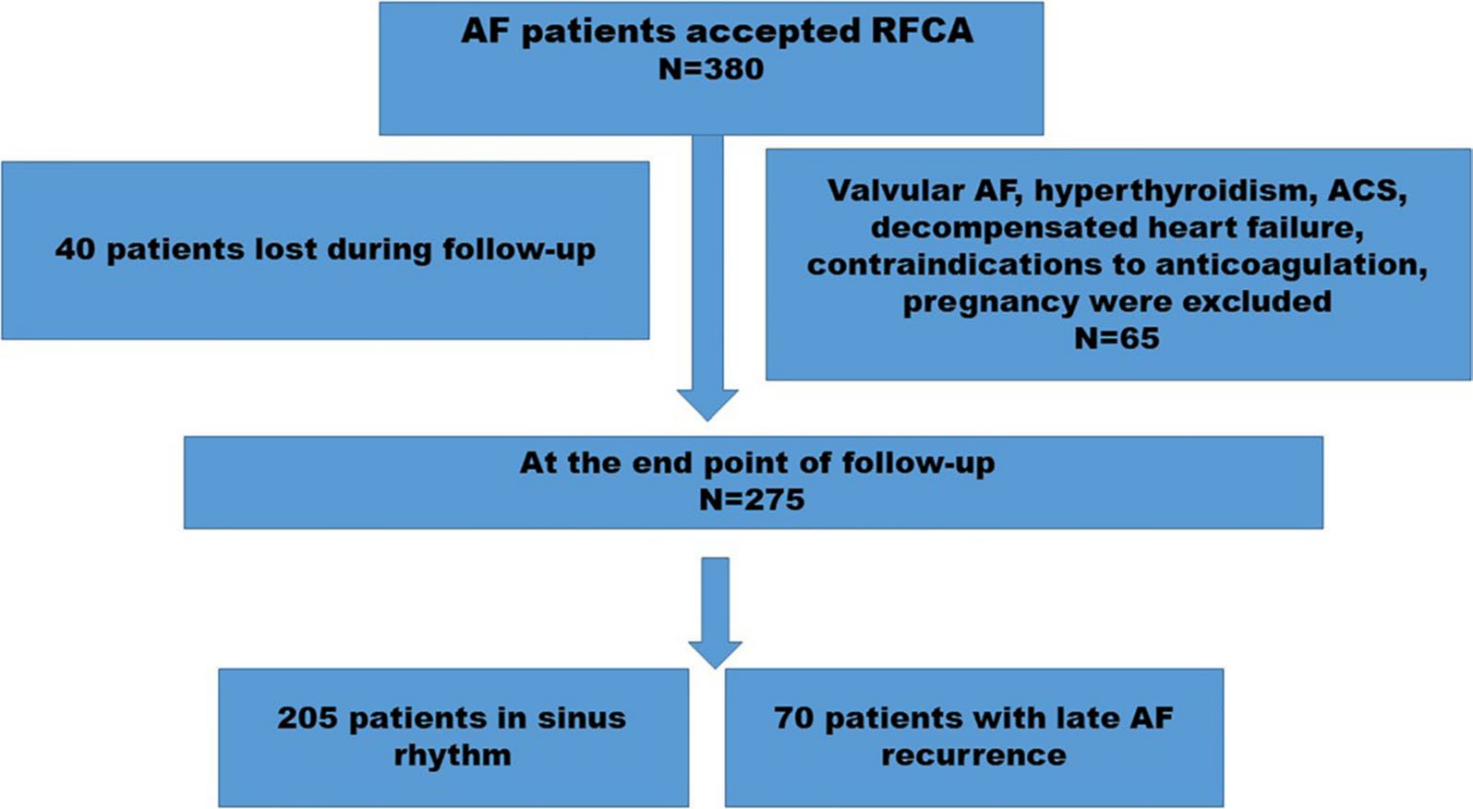
The flow chart of the present study

**Fig. 2:**
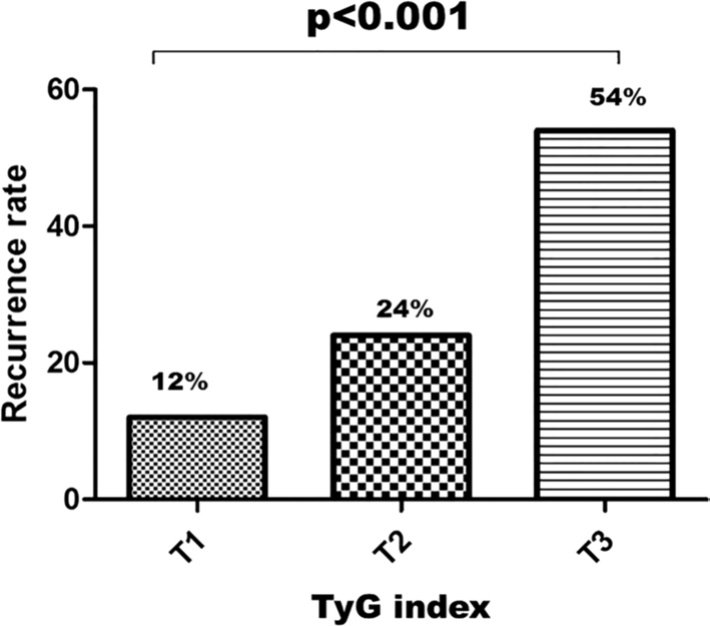
Percentage of the patients developing late AF recurrence post-ablation stratified by tertiles of pre-ablation triglyceride-glucose (TyG) index

Among 380 consecutive non-diabetic patients with AF who underwent radiofrequency ablation, 65 patients who met the major exclusion criteria and 40 patients lost to follow-up were excluded (Fig. 1). Consequently, a total of 275 AF patients (29.1% persistent AF and 70.9% paroxysmal AF) were entered into the analysis. The baseline demographic characteristics, demographical laboratory data, and procedural details of both cohorts are summarized in Table 1. The mean patient age was 57.32 ± 9.57 years, and 69.4% were male. After a median follow-up time of 26.1 months, late recurrence of AF was observed in 70 patients (25.5%). We observed late AF recurrence in 24 patients with persistent AF and 46 patients with paroxysmal AF. As was shown in Table 1, patients with late AF recurrence exhibited larger LA diameter (LAD) (42.4 ± 4.6 versus 37.7 ± 4.1 mm; *p* = 0.001), longer AF duration (79.43 ± 65.7 versus 60.20 ± 58.27 months; *p* = 0.04), older age( 64.38 ± 8.04 versus 55.07 ± 8.93 years; *p* < 0.001), higher number of patients with long-standing persistent AF ( 21.4% versus 8.3%, *p* = 0.010), more patients with early AF recurrence ( 70% versus 12.7%; *p* = 0.001), more patients with hypertension (62.8% versus 46.3%; *p* = 0.02),a higher prevalence of amiodarone treatment (54.3% versus 17.6%; *p* = 0.01), higher CHA_2_DS_2_-VASc score (*p* = 0.02), as well as higher APPLE (*p* = 0.01) and DR-FLASH (*p* = 0.01) scores, compared to patients without late AF recurrence (Table 1). Moreover, the mean value of the TyG index (9.42 ± 0.6 versus 8.68 ± 0.70; *p* < 0.001), high sensitivity C-reactive protein (hs-CRP) (*p* < 0.001), and N-terminal pro B-type natriuretic peptide (NT-proBNP) (483 ± 411 versus 237 ± 205 pg/mL; *p* = 0.005) were significantly greater in patients with late AF recurrence compared to those without late AF recurrence (Table 1). Furthermore, the patients were stratified into three groups according to the value of pre-ablation TyG, as described above, and subgroup analysis showed that patients in T3 group ( tertile 3) had a higher rate of late AF recurrence than those in T1 group ( tertile 1) (54% versus 12%, *p* < 0.001) (Fig. 2). Likewise, patients with a higher TyG index (tertile 3) tended to be older (60.6 ± 10.4 versus 55.1 ± 7.5 years; *p* = 0.001), had elevated BMI (26.3 ± 3.1versus 25.2 ± 2.0 kg/m^2^, *p* = 0.027), enlarged LAD (41.9 ± 4.8 versus 38.1 ± 4.7 mm; p = 0.035), higher hs-CRP level ( 4.8 ± 4.9 versus 2.4 ± 1.6 mg/L; *p* = 0.001), higher NT-proBNP level ( 368.2 ± 379.0 versus 245.7 ± 282.7 pg/mL; *p* = 0.001), higher APPLE score (*p* = 0.01), as well as higher DR-FLASH (*p* = 0.01) and CHA_2_DS_2_-VASc (*p* = 0.025) scores, compared to those in the first tertile (tertile 1**)** (Table 2).

**Table 1:** Clinical characteristics of study population

Parameters	Overall population (n = 275)	Late AF recurrence (+) (n = 70)	Late AF recurrence (−) (n = 205)	*P *value
Age, years (SD)	57.32 ± 9.57	64.38 ± 8.04	55.07 ± 8.93	*p* < 0.001
Male, sex, n (%)	191 (69.4%)	53 (75.7%)	138 (67.3%)	*p* = 0.092
Paroxysmal AF, n (%)	195 (70.9%)	46 (65.7%)	149 (72.7%)	*p* = 0.080
Persistent AF, n (%)	80 (29.1%)	24 (34.3%)	56 (27.3%)	*p* = 0.030
Long-standing Pers AF, n (%)	32 (11.6%)	15 (21.4%)	17 (8.3%)	*p* = 0.010
AF duration, months (SD)	63.03 ± 60.25	79.43 ± 65.7	60.20 ± 58.27	*p* = 0.040
BMI, kg/m^2^	25.7 ± 2.9	25.9 ± 2.6	25.4 ± 3.0	*p* = 0.144
CAD history, n (%)	36 (13.1%)	11 (15.7%)	25 (12.2%)	*p* = 0.063
SAS history, n (%)	37 (13.5%)	11 (15.7%)	26 (12.7%)	*p* = 0.515
Hypertension, n (%)	139 (50.5%)	44 (62.8%)	95 (46.3%)	*p* = 0.020
Previous stroke/TIA, n (%)	13 (4.7%)	3 (4.3%)	10 (4.9%)	*p* = 0.070
Amiodarone use, n (%)	74 (26.9%)	38 (54.3%)	36 (17.6%)	*p* = 0.010
Alcohol intake, n (%)	62 (22.5%)	19 (27.1%)	43 (21.0%)	*p* = 0.090
Current smoking, n (%)	79 (28.7%)	20 (28.6%)	59 (28.8%)	*p* = 0.345
Echocardiography				
LA diameter (AP), mm	38.4 ± 4.68	42.4 ± 4.60	37.7 ± 4.10	*p* = 0.001
LVEF (%)	63.90 ± 5.2	63.66 ± 5.0	64.20 ± 5.2	*p* = 0.907
Laboratory findings				
NT-proBNP, pg/mL	271 ± 254	483 ± 411	237 ± 205	*p* = 0.005
hs-CRP, mg/L	2.29 (1.57–3.15)	3.58 (1.75–5.27)	2.15 (1.47–2.90)	*p* < 0.001
Uric acid,umol/L	340.8 ± 82.8	349.2 ± 79.6	339 ± 81.5	*p* = 0.356
HCy, μmol/L	17.8 ± 2.3	18.3 ± 2.5	16.9 ± 1.9	*p* = 0.068
FPG (mmol/L)	5.5 (5.1–6.2)	6.0 (5.4–6.4)	5.1 (4.7–5.8)	*p* = 0.008
HbA1_C_, %	5.1 ± 0.2	5.1 ± 0.3	5.1 ± 0.1	*p* = 0.352
TC, mmol/L	4.33 ± 1.03	4.35 ± 1.05	4.31 ± 1.01	*p* = 0.125
Non-HDL, mmol/L	3.13 ± 1.05	3.15 ± 1.09	3.11 ± 1.03	*p* = 0.218
LDL-C, mmol/L	2.31 ± 0.43	2.50 ± 0.32	2.20 ± 0.50	*p* = 0.108
HDL-C, mmol/L	1.21 ± 0.30	1.23 ± 0.40	1.18 ± 0.22	*p* = 0.125
TG, mmol/L	1.78 (1.32–2.29)	2.65 (2.09–3.52)	1.65 (1.15–2.18)	*p* = 0.006
Creatinine, μmol/L	79.5 ± 15.7	81.7 ± 15.0	78.7 ± 15.9	*P* = 0.533
TyG index	8.87 ± 0.8	9.42 ± 0.6	8.68 ± 0.70	*p* < 0.001
Clinical scoring point				
EHRA score	2 (1–3)	2 (1–3)	2 (1–3)	*p* = 0.236
CHA_2_DS_2_-VASc score	1 (0–2)	2 (1–3)	1 (0–2)	*p* = 0.020
HAS-BLED score	1 (0–1)	1 (0–1)	1 (0–1)	*p* = 0.219
DR-FLASH score	2 (1–2)	3 (2–4)	1 (1–2)	*p* = 0.010
APPLE score	1 (0–1)	2 (1–3)	1 (0–1)	*p* = 0.010
Early recurrence	75 (27%)	49 (70%)	26 (12.7%)	*p* = 0.001

Data were expressed as mean ± SD, median with 25th and 75th percentile or n (%)

*AF* atrial fibrillation, *BMI* body mass index, *NT-proBNP* N-terminal B-type natriuretic peptide, *HCy* homocysteine, *HbA1*_*C*_ glycosylated hemoglobin, *CAD* coronary artery disease, *SAS* sleep apnea syndrome, *TC* total cholesterol, *LDL-C* low-density lipoprotein cholesterol, *HDL-C* high density lipoprotein cholesterol, *TG* triglycerides, *FPG* fasting plasma glucose, *TyG index* triglyceride-glucose index, *hs-CRP* high-sensitivity C-reactive protein, *LVEF* left ventricular ejection fraction, *LAD* left atrial diameter, *TIA* transient ischemic attack, *Early recurrence* atrial arrhythmia recurrence within three-month blanking period. *EHRA score* EHRA symptom score suggested by European Heart Rhythm Association. *HAS-BLED score* hypertension, abnormal liver/renal function, stroke, bleeding history or predisposition, labile INR, elderly, drugs/alcohol concomitantly

**Table 2:** Clinical characteristics of AF patients according to tertiles of TyG index

Parameters	Tertile 1 n = 131	Tertile 2 n = 77	Tertile 3 n = 67	*P *value
Age, years (SD)	55.1 ± 7.5	57.1 ± 10.6	60.6 ± 10.4	0.001
Male, sex, n (%)	85 (64.8%)	55 (71.4%)	51 (76.1%)	0.395
Paroxysmal AF, n (%)	91 (69.5%)	56 (72.7%)	48 (71.6%)	0.654
AF duration, months (SD)	62.3 ± 60.4	67.9 ± 50.9	69.8 ± 62.8	0.168
BMI, kg/m^2^	25.2 ± 2.0	25.7 ± 3.2	26.3 ± 3.1	0.027
CAD history, n (%)	15 (11.4%)	12 (15.6%)	9 (13.4%)	0.096
Hypertension, n (%)	70 (53.4%)	41 (53.2%)	28 (41.8%)	0.068
SAS history, n (%)	16 (12.2%)	11 (14.3%)	10 (14.9%)	0.218
Previous stroke/TIA, n (%)	6 (4.6%)	4 (5.2%)	3 (4.5%)	0.452
Echocardiography				
LAD, mm	38.1 ± 4.7	39.0 ± 4.5	41.9 ± 4.8	0.035
LVEF (%)	64.0 ± 5.3	63.8 ± 5.1	64.5 ± 5.7	0.674
Biochemistry				
NT-proBNP, pg/mL	245.7 ± 282.7	275.3 ± 325.0	368.2 ± 379.0	0.001
hs-CRP, mg/L	2.4 ± 1.6	3.5 ± 3.8	4.8 ± 4.9	0.001
Uric acid,umol/L	332.5 ± 80.5	338.3 ± 81.2	346.5 ± 79.5	0.352
HCy, μmol/L	16.2 ± 1.5	18.2 ± 2.1	18.6 ± 2.2	0.115
Risk scoring system				
CHA_2_DS_2_-VASc score	1 (1–2)	1 (1–2)	2 (1–3)	0.025
APPLE score	1 (0–1)	1 (1–2)	2 (1–3)	0.010
DR-FLASH score	1 (0–2)	2 (1–2)	3 (2–4)	0.010

Data were expressed as mean ± SD, median with 25th and 75th percentile or n (%)

*AF* atrial fibrillation, *BMI* body mass index, *NT-proBNP* N-terminal B-type natriuretic peptide, *HCy* homocysteine, *CAD* coronary artery disease, *SAS* sleep apnea syndrome, *TyG index* triglyceride-glucose index, *hs-CRP* high-sensitivity C-reactive protein, *LVEF* left ventricular ejection fraction, *LAD* left atrial diameter, *TIA* transient ischemic attack

### Comparison of TyG index

**Fig. 3:**
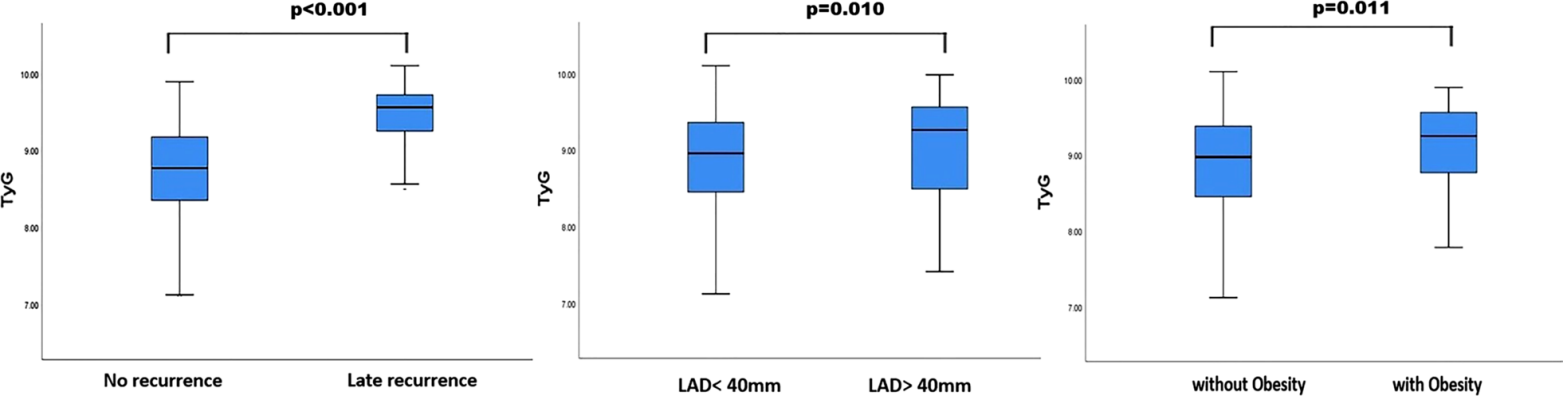
Comparison of triglyceride-glucose index. Box plots represent median levels with 25th and 75th percentiles of the value of TyG index

As was shown in Fig. 3, the TyG index was significantly higher in patients with late AF recurrence compared to patients without late AF recurrence (medians: 9.55 vs 8.75; *p* < 0.001). Moreover, the TyG index was elevated in patients with LAD > 40 mm, compared to patients with LAD < 40 mm (medians: 9.26 vs 8.95; *p* = 0.010). Similarly, the TyG index was elevated in the obese patients compared to non-obese patients (medians: 9.25 vs 8.97; *p* = 0.011).

### Prediction of late AF recurrence using clinical variables

**Fig. 4:**
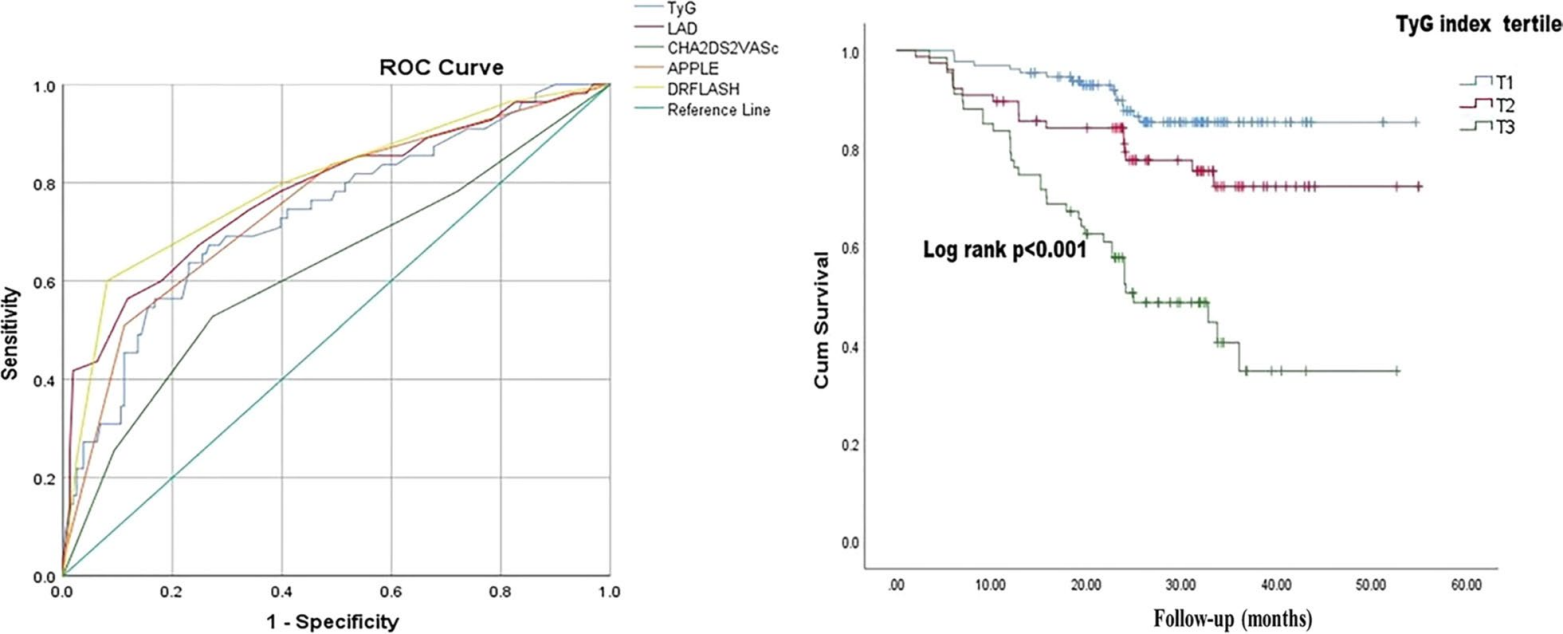
Receiver operating characteristic curve (ROC) of triglyceride-glucose index for predictor of late recurrence of atrial fibrillation after RFCA; Event-free survival analyses according to three tertiles of pre-ablation TyG index

Univariate Cox proportional hazards regression analysis showed that older age (> 65 years), LA diameter, AF type (persistent AF), AF history > 5 years, early AF recurrence, TyG index, LDL-C level, hs-CRP level, NT-proBNP level, CHA_2_DS_2_-VASc score, APPLE score and DR-FLASH score, were significantly associated with late AF recurrence ( all of the variables, *p* < 0.05) (Table 3). Multivariate Cox regression analysis confirmed that TyG index (HR:2.015, 95% CI: 1.408–4.117, *p* = 0.009), LA diameter (HR:3.514, 95% CI: 2.083–5.929, *p* = 0.001), older age(> 65 years) (HR: 1.165,95% CI: 1.013–1.340, *p* = 0.032), early AF recurrence (HR: 1.093, 95% CI: 1.001–1.193, *p* = 0.042), APPLE score (HR: 1.697, 95% CI: 1.116–2.581, *p* = 0.010) and DR-FLASH score (HR: 1.387, 95% CI: 1.052–1.830, *p* = 0.021), were significantly associated with late AF recurrence (Table 3). According to the ROC curve analysis, the TyG index was a significant predictor of late AF recurrence (AUC = 0.737, 95% CI: 0.657–0.816; *p* < 0.001). Additionally, LAD, CHA_2_DS_2_-VASc score, APPLE score, and DR-FLASH score were also significant predictors of late AF recurrence after RFCA (LAD: AUC = 0.780, 95%CI:0.703–0.857, *p* < 0.001;APPLE score: AUC = 0.752, 95% CI:0.675–0.830, *p* < 0.001; DR-FLASH score: AUC = 0.797, 95% CI:0.723–0.871, *p* < 0.001; CHA_2_DS_2_-VASc score: AUC = 0.624, 95% CI:0.533–0.715, *p* = 0.006, respectively) (Fig. 4). The cutoff value for the TyG index was 9.24 based on the ROC analysis, and the corresponding sensitivity and specificity were 89.1% and 57.3%, respectively. Kaplan–Meier analyses revealed that patients in highest tertile of TyG index (T3) presented lower event-free survival, compared to those in the first tertile (p < 0.001 by log-rank test, Fig. 4). In this case, the TyG index may be a reliable predictor for late AF recurrence after RFCA, similar to traditional risk factors, such as old age, LAD, APPLE, and DR-FLASH scores.

**Table 3:** Univariate and multivariate Cox proportional hazards regression analysis of late AF recurrence

Variables	Univariate Cox regression		Multivariate Cox regression	
	HR (95%CI)	*p*	HR (95%CI)	*p*
Clinical parameters				
Age > 65 years	1.693 (1.238–2.316)	< 0.001	1.165 (1.013–1.340)	0.032
Male gender	0.683 (0.301–1.552)	0.360		
Hypertension	1.105 (0.581–2.196)	0.074		
BMI > 28 kg/m^2^	1.251 (0.175–1.151)	0.075		
hs-CRP	1.072 (1.028–1.118)	0.001	1.135 (0.921–1.325)	0.065
NT-proBNP, pg/mL	1.015 (1.007–1.025)	0.001	1.038 (0.881–2.705)	0.121
Uric acid, μmol/L	1.323 (0.912–1.192)	0.733		
LDL-C, mmol/L	2.167 (1.165–4.030)	0.020	1.938 (0.644–5.838)	0.239
HCy, μmol/L	1.702 (0.977–2.965)	0.061		
Creatinine, μmol/L	1.013 (0.996–1.030)	0.135		
TyG index	1.512 (1.220–1.872)	0.005	2.015 (1.408–4.117)	0.009
Alcohol intake	1.451 (0.744–2.95)	0.264		
PersAF	1.103 (1.005–1.210)	0.040	1.172 (0.635–1.819)	0.235
Duration of AF history > 5 years	1.003 (1.000–1.006)	0.029	1.079 (0.642–1.825)	0.775
EHRA score	0.938 (0.563–1.563)	0.805		
CHA_2_DS_2_-VASc score	1.464 (1.126–1.903)	0.003	1.135 (0.977–1.292)	0.055
APPLE score	3.479 (2.442–4.957)	< 0.001	1.697 (1.116–2.581)	0.010
DR-FLASH score	2.309 (2.004–2.835)	< 0.001	1.387 (1.052–1.830)	0.021
Echocardiographic parameters				
LVEF	0.984 (0.937–1.033)	0.515		
LAD	6.477 (3.943–10.639)	< 0.001	3.514 (2.083–5.929)	0.001
Early AF recurrence	2.871 (1.809–4.556)	< 0.001	1.093 (1.001–1.193)	0.042

*AF* atrial fibrillation, *BMI* body mass index, *TyG index* triglyceride-glucose index, *HCy* homocysteine, *NT-proBNP* N-terminal B-type natriuretic peptide, *LDL-C* low-density lipoprotein cholesterol, *LAD* left atrial diameter, *LVEF* left ventricular ejection fraction, *Early AF recurrence* AF recurrence during the blanking period, *hs-CRP* high-sensitivity C-reactive protein

### Correlations between TyG index and key cardiac variables and blood biomarkers

**Fig. 5:**
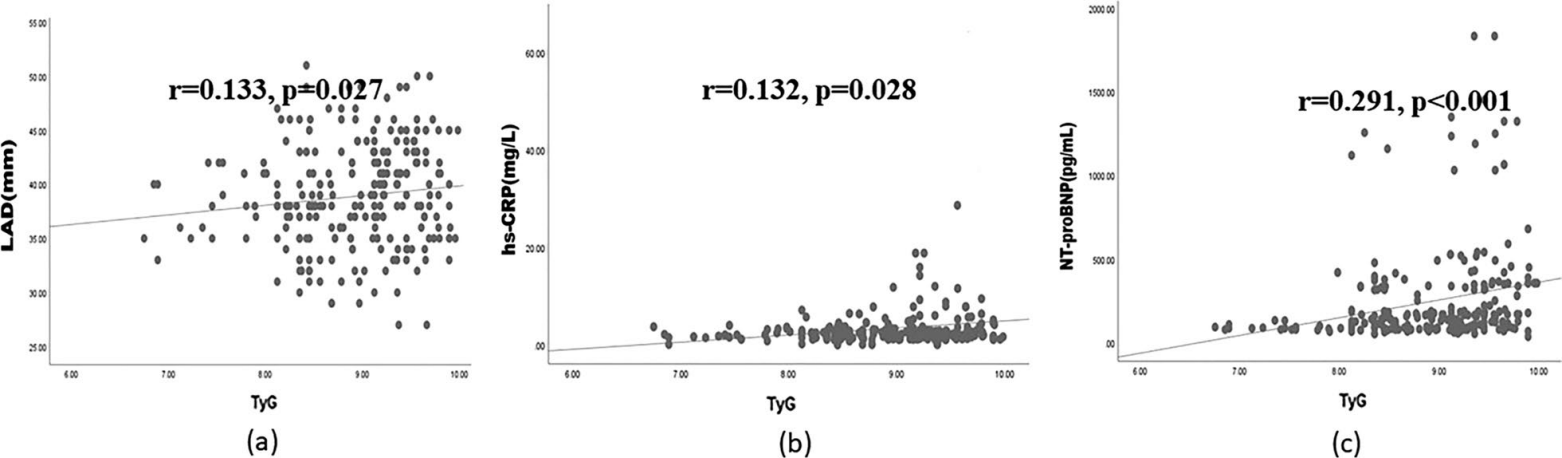
Correlation between LAD, hs-CRP, NT-proBNP and pre-ablation TyG index. **a** TyG index is positively correlated with LAD (r = 0.133, *p* = 0.027); **b** TyG index is positively correlated with hs-CRP level (r = 0.132, *p* = 0.028); **c** TyG index is positively correlated with NT-proBNP level (r = 0.291, *p* < 0.001)

In Spearman correlation analyses, an elevated TyG index was positively correlated with LAD (r = 0.133, *p* = 0.027), hs-CRP (r = 0.132, *p* = 0.028) and NT-proBNP (r = 0.291, *p* < 0.001) (Fig. 5) in non-diabetic patients. However, the correlation coefficients were low, and studies with larger sample sizes are required to validate our results.

### Value of the TyG index according to prognostic risk scoring system

**Fig. 6:**
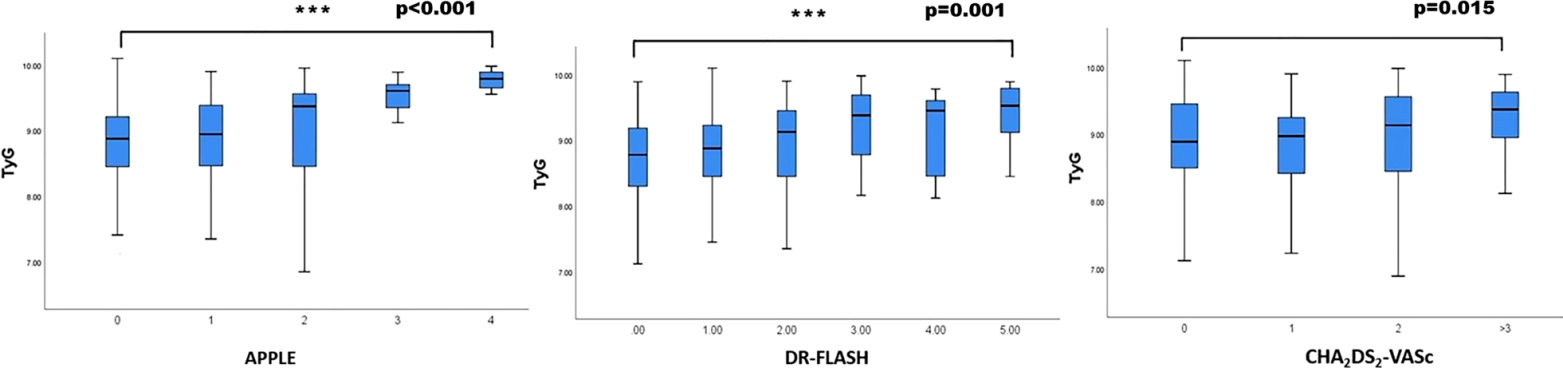
Box plot representing the median value of pre-ablation TyG index at increasing APPLE, DR-FLASH and CHA_2_DS_2_-VASc scores. To compare the value of TyG index according to each of the APPLE, DR-FLASH and CHA_2_DS_2_-VASc scores, ANOVA test was used. ***p < 0.001: the median value of TyG index in the APPLE score of 4 points versus APPLE score of 0 points; ***p = 0.001: the median value of TyG index in the DR-FLASH score of 5 points versus DR-FLASH score of 0 points; the median value of TyG index in the CHA_2_DS_2_-VASc score of > 3 points versus CHA_2_DS_2_-VASc score of 0 points indicated *p* = 0.015

In this study, we found that various prognostic risk scoring systems (APPLE, DR-FLASH and CHA_2_DS_2_-VASc scores) were closely associated with late AF recurrence after RFCA. Furthermore, we investigated the TyG index distribution according to those risk scoring systems. We found that the value of TyG index significantly increased as the mentioned scores elevated: (i) APPLE score (median TyG index 9.65 in APPLE score of 4 points versus 8.80 in APPLE score of 0 points; *p* < 0.001); (ii) DR-FLASH score: median TyG index 9.52 in DR-FLASH score of 5 points versus 8.70 in DR-FLASH score of 0 points; *p* = 0.001); iii) CHA_2_DS_2_-VASc score: median TyG index 9.36 in CHA_2_DS_2_-VASc score > 3 points versus median 8.89 in CHA_2_DS_2_-VASc score of 0 points; *p* = 0.015) (Fig. 6).

## Discussion

In the present study, we assessed the predictive ability of the TyG index to estimate late AF recurrence after RFCA and identified an AUC of 0.737 (95% CI: 0.657–0.816; *p* < 0.001) in non-diabetic patients.

Additionally, we investigated the relationship between the TyG index and LA diameter (LAD), prognostic risk scores, and other key biomarkers. The results revealed that the TyG index levels increased with increasing APPLE, DR-FLASH and CHA_2_DS_2_-VASc scores. Moreover, the TyG index was associated with conventional risk factors for AF such as LAD, hs-CRP and NT-proBNP.

IR refers to the inability of peripheral tissues to correctly use endogenous insulin for maintaining glucose homeostasis in the body [15, 16]. IR is associated with LA remodeling and AF development, even before diabetes [17, 18, 20]. IR may predispose patients to AF progression by increasing LA size or impairing left ventricular diastolic function [18, 20, 21]. Patients with type 2 diabetes usually experience IR and pre- diabetes stages, although these stages are usually not diagnosed in non-diabetic adults [12, 20]. Metabolic syndrome and IR are common in adults without diabetes, and risk factors related to the development of AF have also been described [20, 21]. Previous investigations have confirmed the pathological mechanism of dominant hyperglycemia-related atrial remodeling and arrhythmia in animal models of impaired glucose tolerance [22, 23]. In an animal mode of IR, IR was associated with various aspects of LA remodeling, including increased oxidative stress injury [24], elevated expression of hyperphosphorylated calcium related proteins, and atrial interstitial fibrosis [22–24]. IR promotes LA structural remodeling and abnormal intracellular calcium homeostasis [18], resulting in increased susceptibility to AF [17]. Increased oxidative stress and inflammation can lead to IR and impaired insulin secretion [24]. Inflammation, IR, and oxidative stress injury are linked to one another and, consequently, result in atrial electrical remodeling [18, 24], LA fibrosis [18], and LA low-voltage areas(LVAs) [25], which are likely the main constituents of AF pathophysiology [25, 26]. Most components of IR, such as glucose tolerance abnormalities, obesity and abnormal lipid levels, were found to have an additive effect on the risk of AF progression, which has been reported to be related to late recurrence of AF after RFCA [15, 17].

The TyG index has been proposed as a reliable, valid, and reproducible surrogate marker for IR [13, 14]. Although the hyperinsulinemic-euglycemic clamp is considered as the current gold standard biomarker for measuring insulin sensitivity, it is a complicated evaluation method with limited clinical applicability [14, 27]. As an alternative biomarker, the TyG index was calculated using more accessible and less costly biochemical parameters associated with many cardiovascular diseases (CVDs) [27]. In addition, the TyG index is closely associated with in vivo IR measured using a hyperinsulinaemic-euglycemic clamp [13, 14]. Cardiometabolic risk factors such as dyslipidemia, hyperglycemia, central obesity, and hypertension, which are independent risk factors for CVDs, were independently associated with the TyG index [27, 28]. Patients with cardiometabolic disorders or IR may be more susceptible to AF recurrence following RFCA [15, 17]. To date, only a limited number of metabolic profiling studies investigating late AF recurrence have been performed. Whether the TyG index is associated with late AF recurrence after RFCA is a topic of intense research interest. In the present study, an elevated pre-ablation TyG index was associated with an increased risk of post-ablation AF recurrence. Additionally, the TyG index was associated with conventional risk factors for AF, such as LAD, hs-CRP and NT-proBNP; however, this needs to be further investigated in larger sample studies.

The CHADS_2_ [10], CHA_2_DS_2_-VASc [10], APPLE [9] and DR-FLASH [11] scores are promising prognostic models for atrial arrhythmia recurrence after RFCA. The CHA_2_DS_2_-VASc scoring system has been validated as a risk stratification model for predicting LVAs and poor rhythm outcomes after RFCA [10]. APPLE and DR-FLASH scores were recently verified as predictors of LA fibrosis and LVAs, which are closely associated with AF progression and reflect the severity of the AF stage [9, 11]. A recent study reported that APPLE and DR‐FLASH scores were associated with LA remodeling or fibrosis [9, 11, 25]. In the present study, we found that the elevated TyG index significantly increased as the APPLE, DR-FLASH and CHA_2_DS_2_-VASc scores increased. This implies that the TyG index may reflect the atrial structural remodeling process and late AF recurrence.

### Limitation

Firstly, this study was a single-center retrospective cross sectional study. Secondly, 12-lead ECG or 48-h Holter monitoring was used for follow-up in this study but it may be underpowered when 7-day Holter monitoring and even implantable loop recorder were widely used as standard monitor technology. Not all the patients received routine Holter monitoring in this study and further studies with continuous rhythm monitoring during long-term follow-up are needed to confirm our findings. Finally, more than 10% of patients were lost in the follow-up and larger sample observational studies are needed to validate our result.

## Conclusion

The findings of this study suggest that an elevated TyG index is independently associated with late AF recurrence after RFCA in non-diabetic patients. Large-sample observational studies are warranted to provide conclusive evidence; however, it may be plausible to conclude that the TyG index may be useful as a biomarker for risk stratification and patient selection for AF ablation, among non-diabetic patients.

## Data Availability

The datasets used and/or analyzed during the current study are available from the corresponding author on reasonable request.

## Abbreviations

TyG index: Triglyceride-glucose index
AF: Atrial fibrillation
BMI: Body mass index
CAD: Coronary artery disease
LDL-C: Low-density lipoprotein cholesterol
TG: Triglycerides
FBG: Fasting blood glucose
NT-proBNP: N-terminal pro B-type natriuretic peptide
IR: Insulin resistance;ECG: electrocardiography
hs-CRP: High-sensitivity C-reactive protein
PV: Pulmonary vein
RFCA: Radiofrequency catheter ablation
LA: Left atrial
LAD: Left atrial diameter
LVAs: Low-voltage areas
ROC: Receiver operating characteristic
AUC: Area under the receiver operating characteristic curve
